# Understanding and Willingness to participate in Dementia Clinical trials in Kenya: An Ethnographic Study

**DOI:** 10.1002/alz70859_099671

**Published:** 2025-12-25

**Authors:** Edna N Bosire, Lucy Wambui Kamau, Chinedu T Udeh‐Momoh, Jasmit Shah, Karen Blackmon, Caroline Kalondu Kiio, Irene B Meier, Vaibhav Narayan, Olivera Nesic‐Taylor, Zul Merali

**Affiliations:** ^1^ Brain and Mind Institute, Aga Khan University, Nairobi, Nairobi Kenya; ^2^ Brain and Mind Institute, Aga Khan University, Nairobi Kenya; ^3^ Division of Public Health Sciences, Wake Forest University School of Medicine, Winston‐Salem, NC USA; ^4^ Aga Khan University, Nairobi Kenya; ^5^ Davos Alzheimers Collaborative, Wayne, PA USA; ^6^ Davos Alzheimer's Collaborative, Wayne, PA USA; ^7^ Aga Khan University, The Brain and Mind Institute, Nairobi Kenya

## Abstract

**Background:**

Advancements in dementia research are in part contingent on participation in clinical research. Clinical trials can help researchers determine if new drugs, devices, or diagnostic procedures for dementia are safe and effective. However, little is known about clinical trials for dementia in resource constrained settings such as Kenya. The aim of our research was to explore people’s understanding of dementia clinical trials, their attitudes and perceptions towards dementia clinical trials and what can be done to enhance enrollments on such clinical trials.

**Method:**

This was an ethnographic study involving 8 focused group discussions (FGDs; age and gender stratified; n=81) in Nairobi to understand people’s perceptions about dementia clinical trials. All FGDs were conducted in both English and Kiswahili each lasting about 90 minutes and took place in Kibera and Mathare informal settlements in Nairobi, Kenya. Data were transcribed verbatim and thematically analyzed by Nvivo version 14 software.

**Result:**

Younger participants had a basic understanding of clinical trials compared to older participants. Participant’s willingness to engage in clinical trials for dementia research varied across age and gender groups, and this was influenced by both individual and collective perceptions. Many participants expressed their willingness to participate if they believed the research would contribute to a greater good, such as helping others, improving healthcare or contributing to medical advancement. However, there was hesitation, driven by fear of side effects, concerns about the integrity of the research process, and religious or cultural beliefs. For future motivation to participate in dementia clinical trials, participants recommended a need to ensure their families were involved in such studies, provide financial incentives and sensitizing community members on potential benefits of being participants in clinical trials.

**Conclusion:**

Findings reported from our study may be useful in developing strategies that can enhance participation in dementia clinical research and advance efforts dedicated to finding effective treatments for dementia in Kenya and other similar contexts

